# Diurnal Cycle of Translaminar Pressure in Nonhuman Primates Quantified With Continuous Wireless Telemetry

**DOI:** 10.1167/iovs.61.2.37

**Published:** 2020-02-25

**Authors:** Jessica V. Jasien, Brian C. Samuels, James M. Johnston, J. Crawford Downs

**Affiliations:** 1 Vision Science Graduate Program, School of Optometry, University of Alabama at Birmingham, Birmingham, Alabama, United States; 2 Department of Ophthalmology and Visual Sciences, School of Medicine, University of Alabama at Birmingham, Birmingham, Alabama, United States; 3 Department of Neurosurgery, School of Medicine, University of Alabama at Birmingham, Birmingham, Alabama, United States

**Keywords:** glaucoma, intraocular pressure, intracranial pressure, translaminar pressure

## Abstract

**Purpose:**

Recent retrospective clinical studies and animal experiments have suggested that cerebrospinal fluid pressure (CSFP) is important in glaucoma pathogenesis. Intraocular pressure (IOP) and CSFP are the driving components of the translaminar pressure (TLP), which directly effects the optic nerve head. This study measured the diurnal cycle of TLP using continuous wireless telemetry in nonhuman primates (NHPs), a common animal model of glaucoma.

**Methods:**

We have developed an implantable wireless telemetry system based on a small piezoelectric pressure transducer with low drift. Unilateral IOP was measured in the anterior chamber of the eye, and intracranial pressure (ICP, a surrogate measure of CSFP) was measured in the brain parenchyma in four awake, behaving NHPs for periods of 22 to 281 days. IOP and ICP telemetry transducers were calibrated with direct pressure measurements in the eye (every 2 weeks) and brain (monthly). TLP was quantified in real time as IOP-ICP, and hourly means of IOP, ICP, and TLP were analyzed.

**Results:**

Results show that mean ICP is significantly higher by an average of 4.8 ± 0.8 mmHg during sleeping hours in NHPs (*P* < 0.01). IOP showed a small but significant nocturnal elevation in two of four animals despite NHPs sleeping upright (*P* < 0.05). TLP was significantly lower during sleep (7.1 ± 0.6 mmHg; *P* < 0.01) than when the animals were awake and active (11.0 ± 0.9 mmHg), driven primarily by the large increase in ICP during sleep.

**Conclusions:**

The 56% increase in TLP during waking hours in NHPs matches the increase in TLP due to postural change from supine to upright reported previously in humans.

Intraocular pressure (IOP) is the only clinically modifiable risk factor for glaucoma, and yet disease development and progression can occur at normal mean IOP. In addition, many patients with ocular hypertension never develop the disease. Evidence suggests that glaucoma is a multifactorial disease, and so an individual's susceptibility is likely related to a combination of factors, including the eye's optic nerve head (ONH) structure and biomechanical properties,^1^^,^^2^ tissue and cellular responses to IOP and cerebrospinal fluid pressure (CSFP),^3^^–^^10^ and ocular perfusion pressure (OPP).^11^^–^^14^ Complicating things even further, racial differences^15^^,^^16^ and changes with age^15^^,^^17^^,^^18^ or exposure to chronic IOP elevation/glaucoma^18^^–^^20^ likely influence these factors as well.^2^^,^^21^

CSFP has been hypothesized as a driving factor in glaucoma, as it provides a counter-pressure to IOP, but only at the ONH.^1^^-^^5^ Although IOP acts on the entire globe and affects ONH biomechanics through both laminar and scleral canal stress and strain, CSFP only acts as a retrolaminar pressure. CSFP effects are therefore limited to the ONH and contained lamina cribrosa.^2^^,^^21^^–^^23^ Given this interplay of pressures acting on the ONH, it is plausible that the translaminar pressure (TLP = IOP-CSFP) is more relevant to glaucoma than either IOP or CSFP alone,^24^ although IOP and CSFP may affect the ONH differently according to some numerical simulations^22^ and experimental studies.^23^ It is also important to note that TLP is different from the TLP gradient (TLPG = (IOP-CSFP)/laminar thickness), which is the pressure gradient acting across the thickness of the lamina cribrosa. TLPG captures additional structural information that may prove relevant to glaucoma. Although snapshot IOP measurements can be performed noninvasively in the clinic, CSFP assessment is generally accomplished through lumbar puncture in the lateral decubitus position. As a result, little is known about either IOP or CSFP dynamics, and continuous measurement of TLP fluctuations may provide insight into the mechanisms underlying glaucoma pathogenesis or progression.

Recent retrospective clinical studies and animal experiments have suggested that higher CSFP (measured with a single lumbar puncture) is protective against glaucoma, and low CSFP increases glaucoma risk.^25^^–^^28^ Prospective studies have found that CSFP decreases with age and is lower in patients with primary open angle glaucoma and normal tension glaucoma than healthy controls or patients with ocular hypertension.^3^^,^^25^^,^^27^^,^^29^^,^^30^ However, TLP estimates in these reports are extrapolated due to CSFP and IOP measurements occurring at different time points, sometimes months apart, and lack any data on TLP dynamics.^3^

Very little is known about TLP dynamics, as characterization of the true, 24-hour TLP exposure in individual eyes has not been possible owing to the lack of accurate continuous measurement of both IOP or CSFP in patients or large animal models of glaucoma, such as nonhuman primates (NHPs). Changes in IOP, CSFP, and TLP occur frequently, and at multiple time scales,^6^^,^^31^^–^^34^ yet it is unknown if these fluctuations impact glaucoma development and/or progression. To address this gap in knowledge, we have developed and validated a fully implantable, wireless telemetry system that can measure IOP, intracranial pressure (ICP, a surrogate measure of CSFP), and arterial blood pressure (BP) continuously in NHPs to elucidate the IOP, ICP, OPP, and TLP dynamics over long periods. We use ICP as a surrogate measure of CSFP, but there may not be perfect fluid pressure communication between ICP in the brain and CSFP behind the eye due to cerebrospinal fluid (CSF) compartmentalization and fibrillar tissue in the subarachnoid space.^35^ This system will be used to study the contributions of these pressures on glaucoma development and progression.^6^^,^^31^ This study was designed to measure the diurnal cycle of TLP using continuous wireless telemetry in NHPs, a common animal model of glaucoma.

## Methods

### Animals

All animals were treated in accordance with the ARVO Statement for the Use of Animals in Ophthalmic and Vision Research under an approved Institutional Animal Care and Use Committee protocol monitored by the University of Alabama at Birmingham. Four male rhesus macaques aged 4.5 to 6.5 years, with no ocular abnormalities were used in this study. All animals were kept on a 06:00–18:00 light-dark cycle and fed at approximately 06:00 and 14:00 daily, with water available ad libitum through a continuous feed. Animal demographics are listed in Table 1.

### Stellar IOP, ICP, and Arterial BP Telemetry System

**Figure 1.**
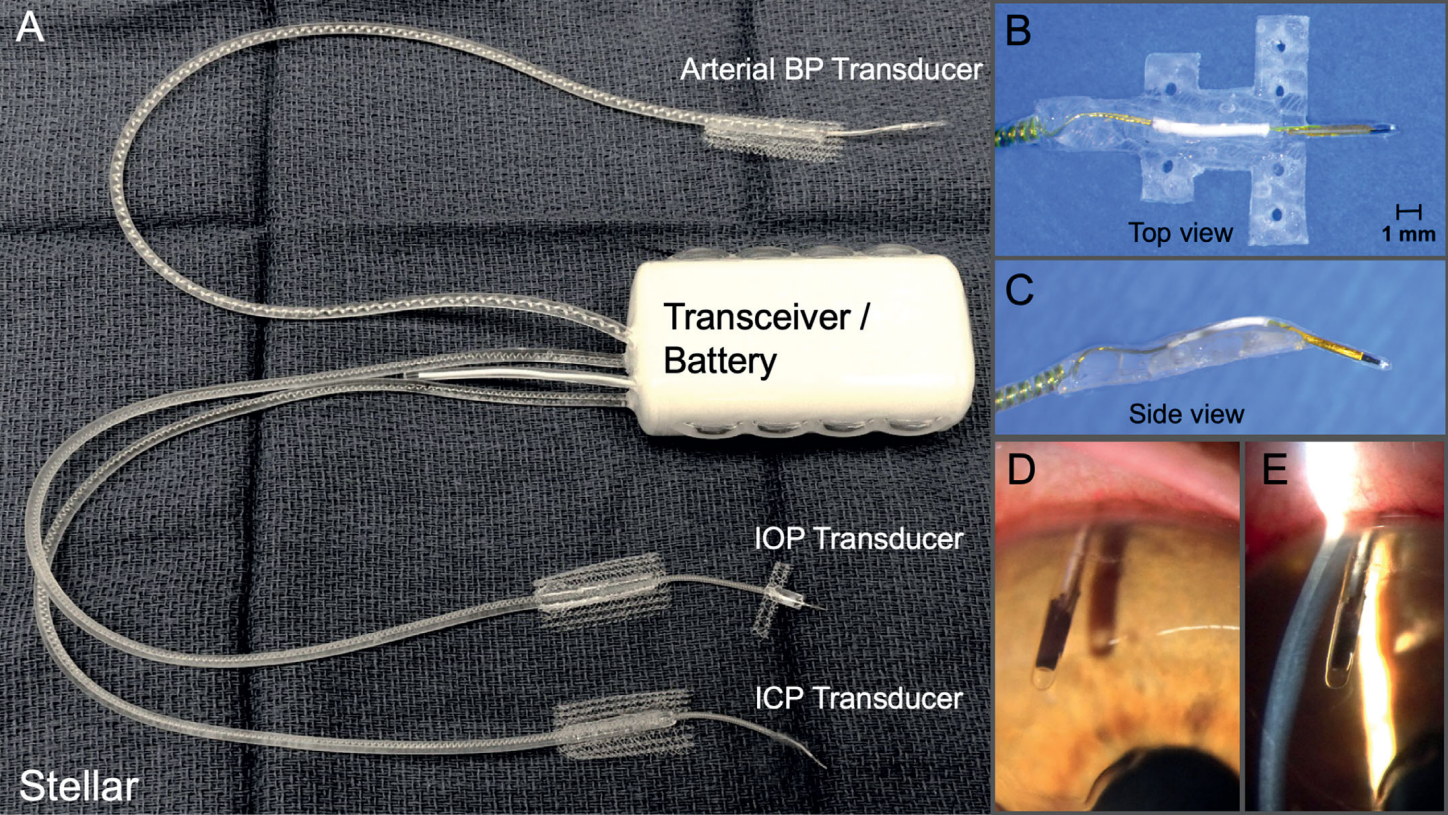
(**A**) TSE-Systems Stellar IOP/ICP/BP total implant system. (**B**) Top view of the IOP transducer and integrated scleral baseplate for affixing the transducer to the eye wall under Tenon's capsule and conjunctiva. (**C**) Side view of the IOP transducer and integrated scleral baseplate. (**D**) En face photograph of the piezoelectric IOP transducer in the anterior chamber. (**E**) Slit lamp photograph of the intraocular placement of the piezoelectric IOP transducer in the anterior chamber relative to the cornea and iris.

**Figure 2.**
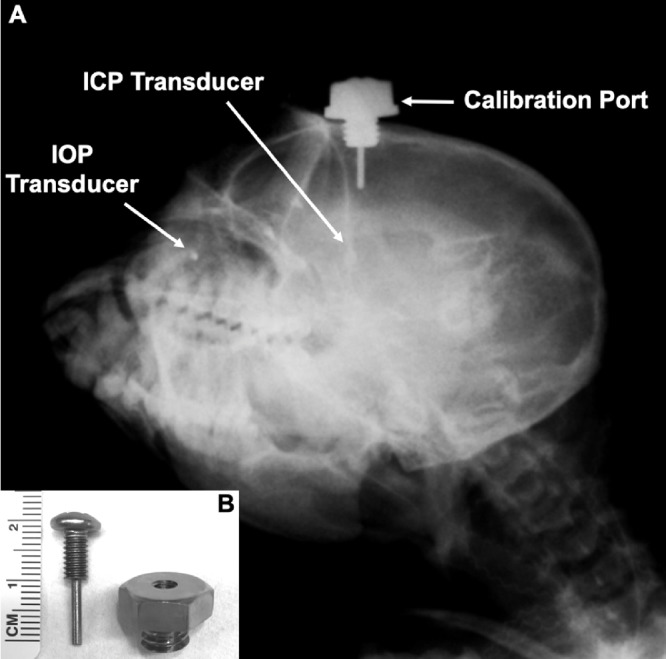
(**A**) Oblique x-ray of an NHP with the Stellar implant (IOP and ICP transducers) and custom indwelling titanium ICP calibration port installed. The IOP transducer is in the right eye and the ICP transducer is in the right lobe of the brain, whereas the calibration port is in the left lobe. The IOP and ICP transducers and bottom of the screw post within the calibration port are all at the same height when the animal's head is in the upright position. (**B**) Photograph of the custom indwelling titanium ICP calibration port, showing the bolt with central screw. Note that the screw is sealed to the bolt with a sterile medical-grade silicone washer when installed.

We have developed an implantable pressure telemetry system based on a small piezoelectric transducer with low drift that accurately measures IOP in the anterior chamber, ICP (as a surrogate for CSFP) in the parenchyma of the brain, and arterial BP in the lumen of a major artery (Fig. 1). The piezoelectric sensor was encased in a 4.5 mm x 0.4 mm diameter borosilicate glass tube, with a port on the side of the tube above the sensor to provide fluid coupling to the sensor itself. For this study, the IOP transducer was placed in the right eye and the ICP transducer was placed in the right frontal lobe, approximately 2.5 cm from the midline of the brain at the same height as the IOP transducer when the animal was upright (Fig. 2). The implantable telemetry system wirelessly records 200 measurements of physiologic pressure per second, for 15 seconds every 150 seconds (10% duty cycle), 24 hours per day.

The implant was placed subcutaneously in an aseptic procedure under general isoflurane inhalant anesthesia (1%–3%) with sustained-release meloxicam and buprenorphine for analgesia, and prophylactic intravenous cefazolin antibiotic. Prior to insertion, the implant unit was gas sterilized in ethylene dioxide. The battery pack/transceiver unit was sutured to underlying musculature between the scapulas with size-0 nylon monofilament through a 6-cm skin incision; the leads and transducers were then routed subcutaneously to the right orbit for IOP, the cranium for ICP, and the left neck or left leg for arterial BP. For IOP transducer placement, a 6.25-mm-diameter hole was drilled in the lateral orbit with a hand drill, and a 1.5-cm fornix incision through the conjunctiva was made parallel to and 4 mm posterior to the limbus in the superotemporal quadrant. The dissection was then carried down through Tenon's to the scleral surface. Blunt dissection of Tenon's from sclera was carried anterior to the surgical limbus and posterior between the lateral and superior rectus muscles. A hemostat was passed posteriorly under the conjunctival fornix incision and through the lateral orbitotomy to grasp the bonneted transducer and pull it into the subconjunctival space. A sclerostomy into the anterior chamber was created with a 25-gauge needle 2 mm posterior to the limbus, and the glass transducer body was inserted into the anterior chamber such that it was equidistant from the cornea and iris. The transducer baseplate was secured to the sclera with 9-0 nylon suture, covered with a half-thickness glycerol-preserved human corneal patchgraft (Alabama Eye Bank, Birmingham, AL, USA), and the incision was closed with 8-0 coated Vicryl suture (Ethicon, Johnson & Johnson, New Brunswick, NJ, USA). The polyethylene lead mesh was secured to the brow ridge adjacent to the orbitotomy with a 3-mm-long self-drilling stainless steel bone screw (50-17903, Stryker Craniomaxillofacial, Kalamazoo, MI, USA). A single subconjunctival injection of 0.1 mL dexamethasone and 0.1 mL cefazolin was administered at the time of surgery. For ICP transducer placement, the cranium was exposed bilaterally through 2.5-cm incisions on the midpupillary line just anterior to the coronal suture. On the right side, a 1.5-mm-diameter craniotomy was made with a twist drill, in the frontal lobe approximately 2.5 cm from the midline. The dura was sharply pierced, and the transducer was inserted intraparenchymally to a depth of 1.5 cm from the skull surface. The polyethylene mesh was secured to the skull with a 3-mm bone screw, and the lead mesh, bone screw, and craniotomy were covered with acrylic dental cement (3M Duralon, St. Paul, MN, USA). On the left side, a 5/16-inch diameter craniotomy was made with a twist drill, in the frontal lobe approximately 2.5 cm from the midline. The dura was sharply pierced, and the custom titanium craniotomy port was threaded into the skull until tight and flush with the bone; the center screw was then installed to a depth of 1.5 cm from the skull surface with a sterile silicone washer in place to prevent CSF egress (Fig. 2). For BP measurement, the femoral or carotid artery was isolated via blunt dissection and a sterile size-0 silk suture was placed under the vessel proximal to the insertion point. The suture was elevated to temporarily stop blood flow for approximately 30 seconds, while the vessel wall was opened with a bent 18-guage needle and the arterial pressure transducer inserted into the vessel lumen. Vetbond cyanoacrylate adhesive (3M, St. Paul, MN, USA) was applied to secure the polyethylene mesh atop the vessel and seal the transducer lead against leakage at the insertion site. All skin incisions were closed with 4-0 PDS II subcuticular suture (Johnson & Johnson, New Brunswick, NJ, USA), and glued superficially with Vetbond. The animals were recovered in their cages and provided warm blankets and a heat lamp for several days following surgery; sustained-release buprenorphine and meloxicam analgesia were administered for 5 to 7 days as needed. The study protocol mandates a minimum 4-week recovery time following surgery before data collection began.

### IOP Calibration

**Figure 3.**
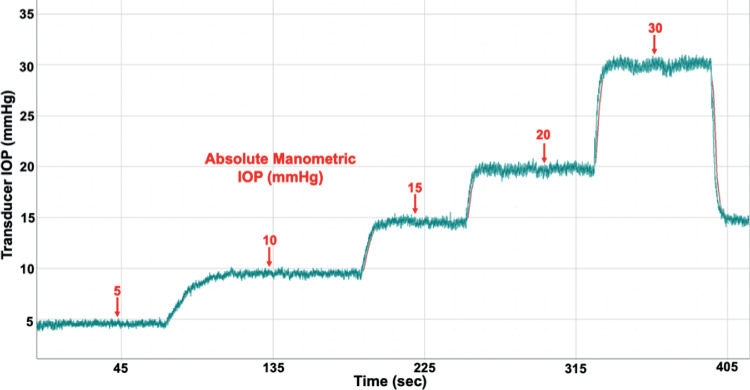
Screenshot of transducer (raw, uncalibrated) and true manometric IOP captured during a representative IOP transducer calibration procedure from 5 to 30 mmHg. Transducer IOP and manometric IOP are approximately 1 mmHg different in this case; IOP transducer drift is accounted for with biweekly calibration, and all IOP data are drift-corrected via software postprocessing.

The IOP telemetry transducer was calibrated every 2 weeks via anterior chamber cannulation manometry with a 27-gauge needle placed through the cornea at the limbus under slit-lamp biomicroscopy, connected to a sterile saline reservoir via sterile infusion set equipped with an in-line digital pressure gage (Model XP2i, Crystal Engineering, San Luis Obispo, CA, USA). Anesthesia was induced using ketamine (3 mg/kg) with dexmedetomidine (50 μg/kg; intramuscular injection) and maintained using inhaled isoflurane (1%–3%) during transducer calibrations. Proparacaine hydrochloride (0.5% ophthalmic solution) was applied to anesthetize the cornea prior to anterior chamber cannulation. All NHPs were kept warm with a warming blanket and systemically monitored for heart rate, SpO_2_, end-tidal CO_2_ volume, electrocardiogram, and temperature during all procedures, with documentation every 15 minutes. To assess the error in the IOP transducer reading, absolute manometric IOP was elevated from 5 to 30 mmHg in 5 to 10 mmHg steps. IOP was allowed to stabilize at each setpoint while telemetric IOP was recorded at 200 Hz (Fig. 3). All IOP data were corrected for signal drift between calibrations using the 15 mmHg value; transducer drift was approximately 2 mmHg a month.

### ICP Calibration

**Figure 4.**
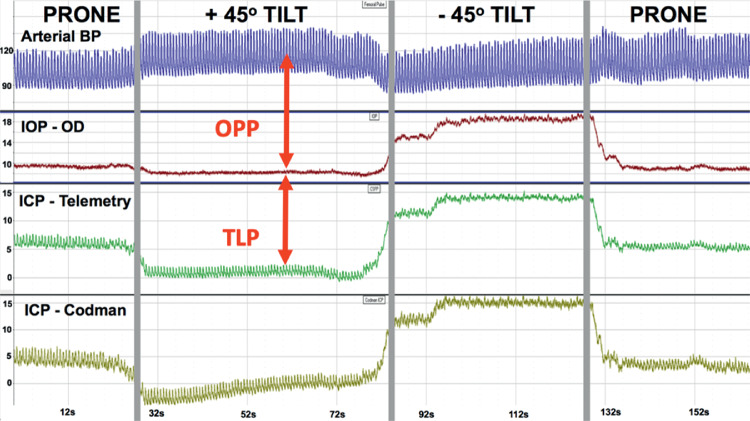
Screenshot of the pressure telemetry signals acquired during a typical ICP transducer calibration procedure in which the NHP is moved from the prone position to positive 45° incline (head up), to negative 45° decline (head down), and back to prone. Arterial BP (blue), IOP in the right eye (red), ICP from Stellar Telemetry (green), and ICP from the Codman microsensor (gold) are recorded at 200 Hz during the calibration procedure; Y-axis scale is mmHg for all signals.

**Figure 5.**
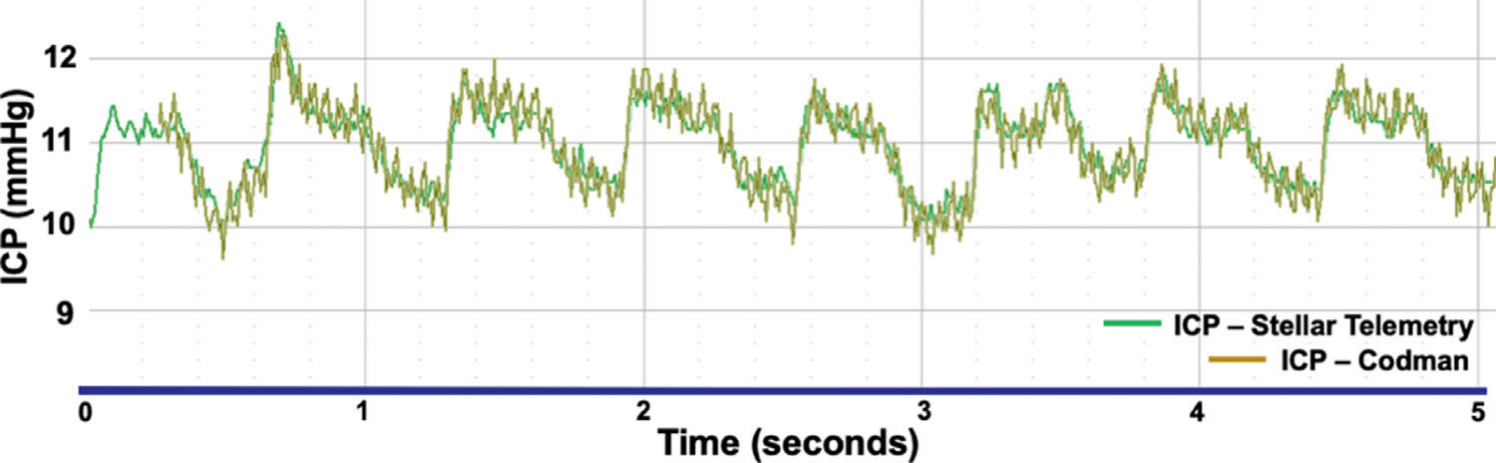
Screenshot of the ICP (mmHg) signals from the calibrated Stellar Telemetry transducer (green) and Codman microsensor (gold) overlaid during an ICP transducer calibration session. The signals are nearly identical in that each system displays the same pulse pressure and amplitude.

The indwelling Stellar ICP transducer was calibrated every 4-weeks using the clinical gold-standard Codman ICP Express microsensor (DePuy Synthes, Raynham, MA, USA) that measured ICP in real time through an indwelling custom titanium port in the cranium that allows access to the left brain parenchyma (Fig. 2). For ICP calibration, the skin was opened above the indwelling cranial port, the central screw was removed, and a Codman microsensor was placed in the brain parenchyma to the depth of the ICP transducer in the adjacent lobe; a sterile silicone rubber washer was used to seal the microsensor strain gauge cable to the interior sidewall of the port bolt to prevent CSF egress and maintain ICP. ICP was then measured by both the indwelling Stellar telemetry transducer and the Codman microsensor, and the values were compared to assess Stellar pressure transducer drift. All Codman data were recorded simultaneously with the Stellar telemetry signals with NOTOCORD-hem data acquisition software (Instem, Stone, Staffordshire, UK) through a custom calibrated amplifier. ICP (telemetry and Codman), IOP, and arterial BP were measured and recorded at 200 Hz, while the NHP was moved from the beginning baseline prone position to +45° (head up) incline position, and –45° (head down) decline position, then back to the prone position (Fig. 4). Each position was held until the pressures of the Stellar ICP transducer and the Codman ICP microsensor were stable. There was remarkable agreement between ICP measured with the Codman microsensor and indwelling Stellar telemetry transducer during these procedures (Fig. 5). The prone position was used as the calibration reference for all calibration sessions in which the Stellar ICP transducer was calibrated against the Codman ICP microsensor to account for signal drift. ICP transducer drift was similar to IOP transducer drift at approximately 2 mmHg per month; IOP and ICP data were calibrated to either manometric or clinical standards, and calibrated TLP was quantified as IOP-ICP (Fig. 4). All pressure data were drift-corrected continuously between calibration procedures via software postprocessing assuming linear drift between calibrations. It is important to note that ICP data were not calibrated in animal 150143 owing to failure of the ICP transducer prior to the second calibration procedure, however, ICP in the remaining three animals was calibrated.

### Data Procurement and Analysis

ICP and IOP data were measured and recorded at 200 Hz for a period of 15 seconds every 150 seconds (10% duty cycle) over a span of 22 to 281 days (depending on the animal) using NOTOCORD-hem data acquisition software. Atmospheric pressure was measured and compensated for in real time. Data were averaged as hourly means over consecutive days to identify a diurnal cycle in IOP, ICP, and TLP. A paired *t*-test was used to compare the IOP, ICP, and TLP in the sleeping (18:00–06:00) and waking (06:00–18:00) periods within each animal. It is important to note that NHP 150143 ICP and TLP values were uncalibrated, but IOP was calibrated. Even though ICP and TLP values from NHP 150143 were uncalibrated, comparing the relative difference between the sleep and wake periods is valid, so these data were reported as well.

## Results

### Intraocular Pressure

**Figure 6.**
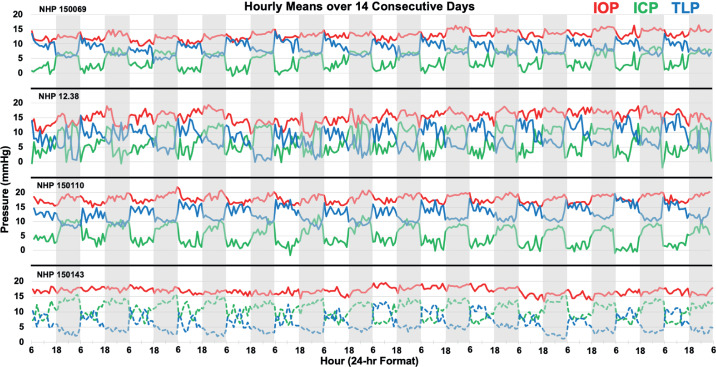
Hourly mean pattern of IOP (red), ICP (green), and TLP (blue) in four NHPs over a randomly selected period of 14 consecutive days. Gray bars indicate the sleeping period (18:00–06:00). Note that ICP (dashed green) and TLP (dashed blue) are uncalibrated in NHP 150143 so absolute pressures are not valid, although relative diurnal differences in pressure are accurate.

**Figure 7.**
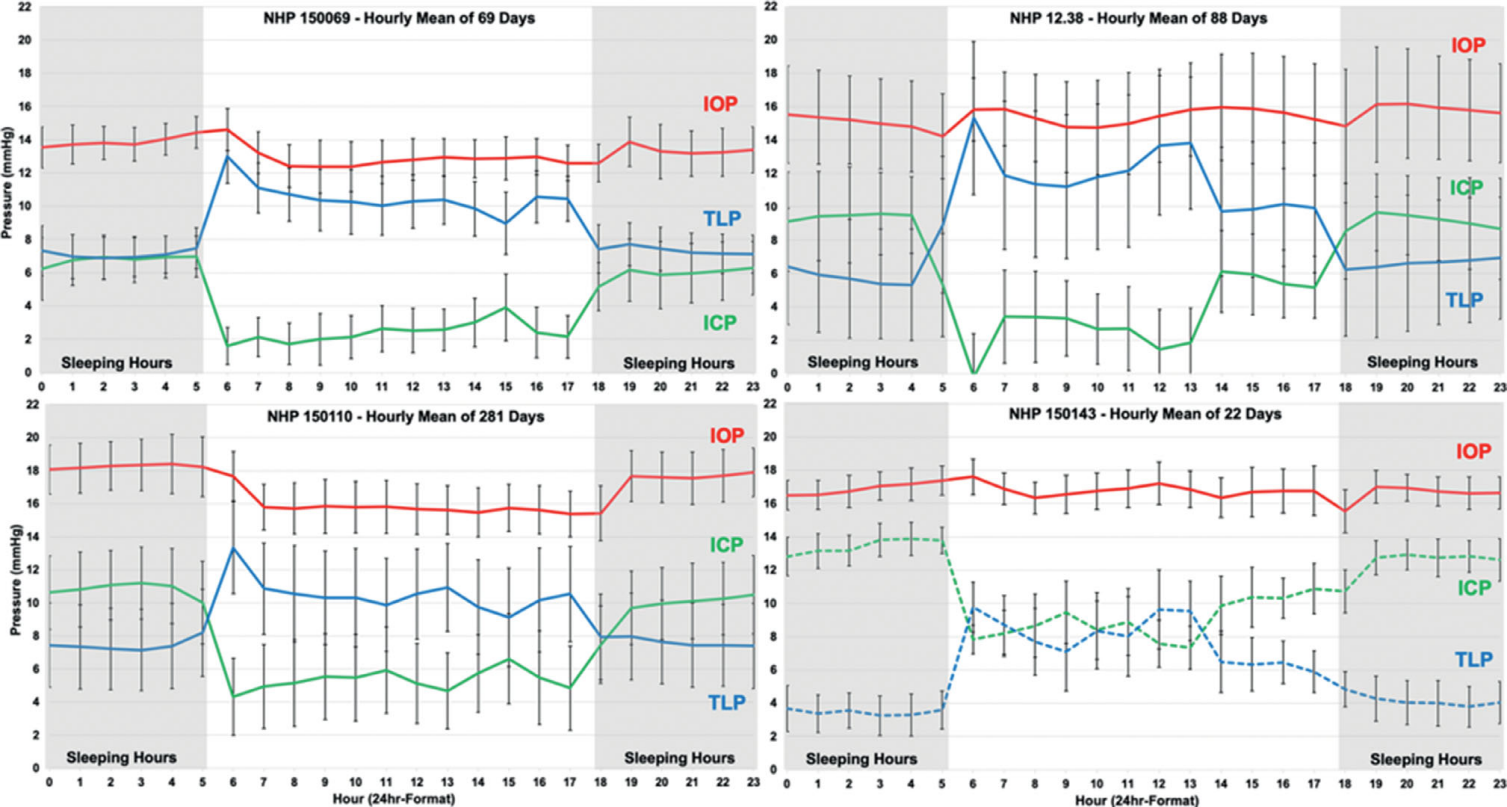
Hourly mean IOP (red), ICP (green), and TLP (blue) across all days in each NHP, plotted on a 24-hour scale. Gray areas indicate the sleeping period (00:00–06:00 and 18:00–00:00). Note that ICP (dashed green) and TLP (dashed blue) are uncalibrated in NHP 150143 so absolute pressures are not valid, although relative diurnal differences in pressure are accurate.

IOP showed significant but small nocturnal elevation of 0.7 to 1.9 mmHg in two of four animals despite NHPs sleeping upright (^1^Table 2). Figure 6 shows the hourly means of IOP, ICP, and TLP in a randomly selected, consecutive, 14-day period for each NHP, in which the gray bars represent the nocturnal sleeping period of 18:00–6:00. A subtle but consistent diurnal cycle in IOP was apparent in animals 150110 and 150069. Figure 7 shows the hourly average values for all pressures across all days collected, and the diurnal cycle in IOP was again apparent in animals 150110 and 150069.

**Table 1.** Animal Demographics

NHP	Age (Years)	Sex	Number of Days
**150069**	4.5	Male	69
**12.38**	6.5	Male	88
**150110**	4.5	Male	281
**150143**	5	Male	22^*^

* IOP is calibrated, but ICP and TLP are uncalibrated.

**Table 2.** Average IOP, ICP, and TLP (Mean ± SD in mmHg) for the Waking (6:00–18:00) and Sleeping (18:00–6:00) Periods Across all Days by NHP

	IOP		ICP		TLP	
NHP	Awake	Sleep	Awake	Sleep	Awake	Sleep
**150069**	12.9 ± 0.6	13.6 ± 0.5^*^	2.4 ± 0.6	6.4 ± 0.6^†^	10.5 ± 0.9	7.2 ± 0.3^†^
**12.38**	15.5 ± 0.4	15.4 ± 0.6	3.4 ± 1.9	8.9 ± 1.2^†^	12.0 ± 1.9	6.5 ± 0.9^†^
**150110**	15.9 ± 0.6	17.8 ± 0.8^†^	5.3 ± 0.6	10.2 ± 1.0^†^	10.5 ± 1.0	7.6 ± 0.3^†^
**150143**	16.8 ± 1.2	16.7 ± 1.0	9.0 ± 1.9	12.9 ± 1.3^†^	7.8 ± 2.3	3.8 ± 1.3^†^

† *P* < 0.01

* *P* < 0.05 indicate significant change between wake and sleep. Note that ICP and TLP values are uncalibrated in NHP 150143 so absolute pressures are not valid, although relative diurnal differences in pressure are accurate.

### Intracranial Pressure

Results show that ICP was 3.9 to 5.5 mmHg greater during sleeping hours (*P* < 0.01) compared with waking hours in all animals (Table 2). In relative terms, ICP was 92%–166% higher during sleeping hours than during waking hours when the animals were active (*P* < 0.01, in the three NHPs for which data were calibrated). A large diurnal cycle in ICP was apparent in all animals in Figures 6 and 7.

### Translaminar Pressure

TLP was significantly higher during the waking period than the sleeping period, by an average of 2.9 to 5.5 mmHg depending on the animal (*P* < 0.01; Table 2). The large magnitude of the diurnal cycle in ICP, which was much larger than the diurnal cycle in IOP, was driving diurnal cycle in TLP. In relative terms, TLP was 38%–85% higher during waking hours than during sleeping hours (*P* < 0.01, in the three NHPs for which data were calibrated). TLP was highest on waking and generally decreases somewhat during the remainder of the waking period (Figs. 6 and 7).

### Discussion

**Figure 8.**
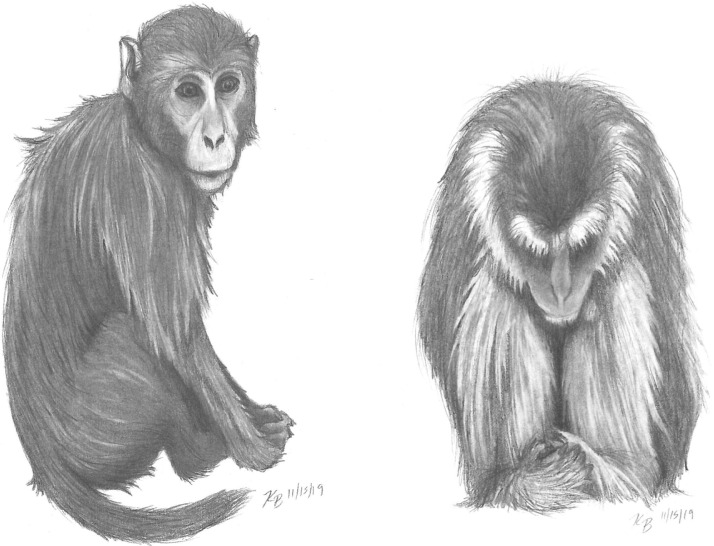
Illustrations of a rhesus macaque in the awake (left) and sleeping (right) postures. (Courtesy Kelsey Browning, with permission.)

We have developed and validated a novel wireless telemetry system capable of continuous monitoring of IOP, ICP (a surrogate for CSFP), and arterial BP dynamics over long periods in NHPs. TLP exhibits a nychthemeral rhythm despite the fact that NHPs sleep sitting up, with TLP showing a 2.9 to 5.5 mmHg increase during waking hours over that measured during sleeping hours. IOP was increased significantly during sleeping hours in two of the four NHPs, but by smaller absolute and relative magnitudes than that seen in ICP and TLP. One striking finding was that ICP increased significantly during sleeping hours in NHPs despite their propensity to sleep upright, which matches the increase in ICP reported in humans that occurs with supine position during sleep. This was unexpected. NHPs sleep with their neck flexed (Fig. 8), and hence we hypothesize that the large increase in ICP and corresponding decrease in TLP during sleeping hours in NHPs is due to increased venous congestion and pressure in the head, associated with neck flexion instead of a shift in the hydrostatic pressure column seen with positional change. One other possible explanation for the elevation of ICP observed during sleep may be elevation of partial pressure of carbon dioxide and concomitant elevated cerebral blood flow due to decreased respiratory rate associated with deep sleep.

There is a strong and consistent diurnal cycle in ICP and TLP in NHPs in which TLP is much higher during waking hours, as seen in all four animals. This pattern matches that reported for humans but is opposite of that reported in rodents. Lin and Liu^36^ reported a circadian variation of ICP and TLP in rats during a 12-hour light-dark cycle in which TLP was significantly higher during sleeping hours, driven by a significant increase in IOP at night. Most importantly, they reported no diurnal variation in ICP in rats.^36^ This is the opposite of what we found in NHPs, in which TLP was significantly lower during sleeping hours, driven primarily by a large diurnal variation in ICP. These stark differences in the diurnal cycle of ICP and TLP in NHPs and rats are likely owing to differences in posture, activity during the light and dark periods (rats are nocturnal), and postural changes in NHPs during sleep. The relative amplitude of the diurnal cycle in TLP reported herein for NHPs (4.2 mmHg or 56% mean increase during waking hours) is remarkably similar to human postural changes associated with the diurnal cycle (8.6 mmHg or 56% mean increase from supine to sitting position,^37^ making the NHP a better model for human TLP dynamics than other animals for which IOP, ICP/CSFP, and TLP data are available.

Unlike TLP, the TLPG (TLPG = (IOP-CSFP)/laminar thickness) is the pressure gradient acting across the thickness of the lamina cribrosa; TLPG takes the laminar structure into account and may be even more relevant to glaucoma than IOP, CSFP, or TLP. Morgan and colleagues^38^ measured TLPG in dogs and found a strong correlation between the TLPG and TLP when the ICP was greater than 0 mmHg; Hou and colleagues^39^ also showed a positive correlation between ICP and retrolaminar tissue pressure above 3 mmHg in dogs, although retrolaminar pressure was constant when ICP was less than 3 mmHg. These studies showed that ICP and retrolaminar pressure are generally similar in dogs, therefore it is reasonable to use ICP as a surrogate for CSFP when quantifying TLP in NHPs, as done in the present study.^38^^–^^40^

This study has the following limitations. First, the study is based on a relatively small sample size of four NHPs that may not represent the full range of TLP in the population. That said, the results were statistically significant because of the large diurnal variation in TLP and the consistent behavior across all four animals. Second, experimental considerations led to a wide range of days of data available across NHPs; again, even though we had as little as 22 days of data available for one animal, the results were consistent across days and so the conclusions are robust. Third, it has been reported that ICP decreases with age in humans,^3^^,^^29^ and the NHPs studied herein are young adults; future studies will be needed to confirm that a similar nocturnal increase in ICP is found in older NHPs. Finally, we use ICP measured in the brain parenchyma as a surrogate for CSFP in the optic nerve sheath. Although there is some evidence that fibrous material in the subarachnoid space surrounding the optic nerve in humans may impede fluid exchange between the brain and retrolaminar optic nerve,^35^ retrolaminar tissue pressure measurements in dogs suggest that retrolaminar CSFP tracks ICP well.^38^^–^^40^ We were careful to position the ICP transducer at the height of the eye to avoid hydrostatic pressure effects. Also, clinical studies have used CSFP measured via lumbar puncture in the lateral decubitus position, which is also remote to the eye and should track ICP. Finally, ICP is generally measured in the third ventricle, which has been shown to correlate with retrolaminar pressure in the lateral decubitus position, and hence CSFP and ICP should generally be interchangeable.^39^^,^^41^

 In future studies, we will measure continuous IOP, ICP, and TLP and identify the basic physiology of TLP with changes in body position. We will also measure lamina cribrosa thickness and report TLP and TLPG patterns in rhesus macaques in vivo for both normal eyes and as glaucoma progresses.

## Conclusions

The data presented herein show that there is a consistent nychthemeral rhythm in TLP across NHPs. This variability, coupled with telemetry, should allow us to test the hypotheses that IOP, ICP, and/or TLP fluctuations contribute independently to glaucoma onset and/or progression.
